# Estimation of nutrient loads with the use of mass-balance and modelling approaches on the Wełna River catchment example (central Poland)

**DOI:** 10.1038/s41598-022-17270-4

**Published:** 2022-07-29

**Authors:** Damian Bojanowski, Paulina Orlińska-Woźniak, Paweł Wilk, Ewa Szalińska

**Affiliations:** 1 AGH University of Science and Technology, A. Mickiewicza Av. 30, 30-059 Krakow, Poland; 2 Institute of Meteorology and Water, Management - National Research Institute, Podleśna 61, 01-673 Warsaw, Poland

**Keywords:** Freshwater ecology, Hydrology, Computational science

## Abstract

Nitrogen and phosphorus budgeting is considered to be a key tool for policy makers and stakeholders when dealing with nutrient contamination issues, however no unified method has been employed in countries affected by this eutrophication problem. The current study offers a detailed insight into the estimations of nutrient loads and their distribution between different sources for a middle-sized agricultural catchment, with the use of two approaches: mass balance (static) and modelling (dynamic). Both methods revealed similar contributions of analysed nutrient sources, although the final estimates in the chosen calculation profile were divergent due to the various reasons related to the methods’ specificity. The advantages and disadvantages of both approaches have been specified in our study, and a hybrid solution on a local and country wide scale has been proposed.

## Introduction

Nitrogen and phosphorus are two key nutrients in aquatic and soil environments, and play an important but somewhat contradicting role in the sustainable development of global and local ecosystems. They are essential for growing crops and maintaining food security, although in many catchments may lead to nutrient overloading as a result of agricultural and industrial activities. As a consequence, the phenomenon of eutrophication is permanently observed, including the majority of surface water bodies, and its intensity and extent is growing at an almost pandemic scale, therefore the term "*new wave of eutrophication*" is currently being used^1^. Consequently, effective nutrient management is one of the main challenges in many countries' development agendas. This issue is also promoted and fostered by many international initiatives and organisations, e.g., the United Nations, “*The nutrient challenge*” or HELCOM’s “*Nutrient reduction scheme*”. To address this challenge, nutrient budgeting, which relies on the estimation of inputs and outputs in a given system, is commonly used to provide a useful tool for policy makers and stakeholders. Despite their long history, nutrient budgets still pose many problems, related mainly to the insufficient availability of monitoring or anthropogenic activity data in a given scale^2^. Although at the same time they have stimulated the development of many complex and advanced modelling tools^3^.

In Europe, where approx. 39% of the total area is used for agricultural production^4^ the monitoring of, i.a., nutrient pollution in riverine systems is conducted, in line with the requirements of the European Water Framework Directive (EU WFD). As for the Baltic Sea catchment, the Commission for the Protection of the Marine Environment of the Baltic Sea Area (HELCOM) has obliged all countries that contribute to its pollution, to balance their nutrient inputs^5^. Since HELCOM has given its members great flexibility in choosing a nutrient balancing method, a great variability of approaches can be observed among these countries. For the estimation of riverine nutrient loads, emission from sources, retention and load distribution, the following approaches have been selected: (i) calculation methods such as the mass-balance method used in Poland, Finland, Latvia, and Germany); (ii) simulation methods with the use of models such as HYPE, SWAT or the EstModel used in Russia, Sweden, Lithuania, and Estonia; iii) combined approaches used e.g. in Denmark^6^.

In Poland, agriculture covers as much as 60% of the country's area^7^ and is considered to be the main pressure on surface waters in many catchments^8^. Despite multiple legislative and managerial efforts in the past and recent years^9–11^ deterioration of water quality resulting from agricultural activities remains the biggest challenge of water management in Poland. To enhance effectiveness of actions focused on reduction of water pollution from these sources and restoring proper water quality status, including protected areas ^12^ the extensive information on pollutant loads and their spatial and temporal distribution is required. Until recently, only a mass balance method has been used in Poland, which allowed to distinguish individual sources of nutrients in the catchment area, but only at a specific time and place (static approach) and based on numerous judgmentally adopted coefficients. Consequently, despite its simplicity and ease of use, the results are difficult to use for effective supervision and countermeasures, especially in a local context.

Limitations of this approach have forced a shift towards modelling (dynamic approach) to give better recognition of nutrient balancing and enable proper identification of problem areas. Despite the fact that nutrient balancing for various catchments using both static and dynamic approaches is frequently reported in many studies^13–16^, no information is available on the results of a direct comparison between these two methods for the same area. Moreover, individual sources in the modelling approach are rarely estimated in the separate scenarios. Therefore, the first goal of our study is to respond to the absence of this knowledge by demonstrating the variability of nutrient loadings from different sources in the very same catchment, using two substantially different methods of nutrient balancing, i.e., mass-balance vs. modelling approach. The mass-balance method (static, load oriented approach) was based on the estimation of nitrogen and phosphorus inputs from the point and of diffuse sources based on the HELCOM guidelines and adopted by Polish PLC-7 methodology^17^ used for reporting nutrient emission in the Baltic Sea Basin area and load inputs to the Baltic Sea. The modelling approach (source oriented), meanwhile, was based on the Macromodel DNS/SWAT and an advanced multi-stage scenario analysis. This comparison has been performed for the agricultural catchment of the Wełna River of central Poland, and enabled visualisation of advantages and disadvantages of both methods. Moreover, as a second goal of our study we propose a hybrid approach to nutrient balancing, which offers multiple benefits on both scales, local and country wide.

## Methods

### Case study area

**Figure 1:**
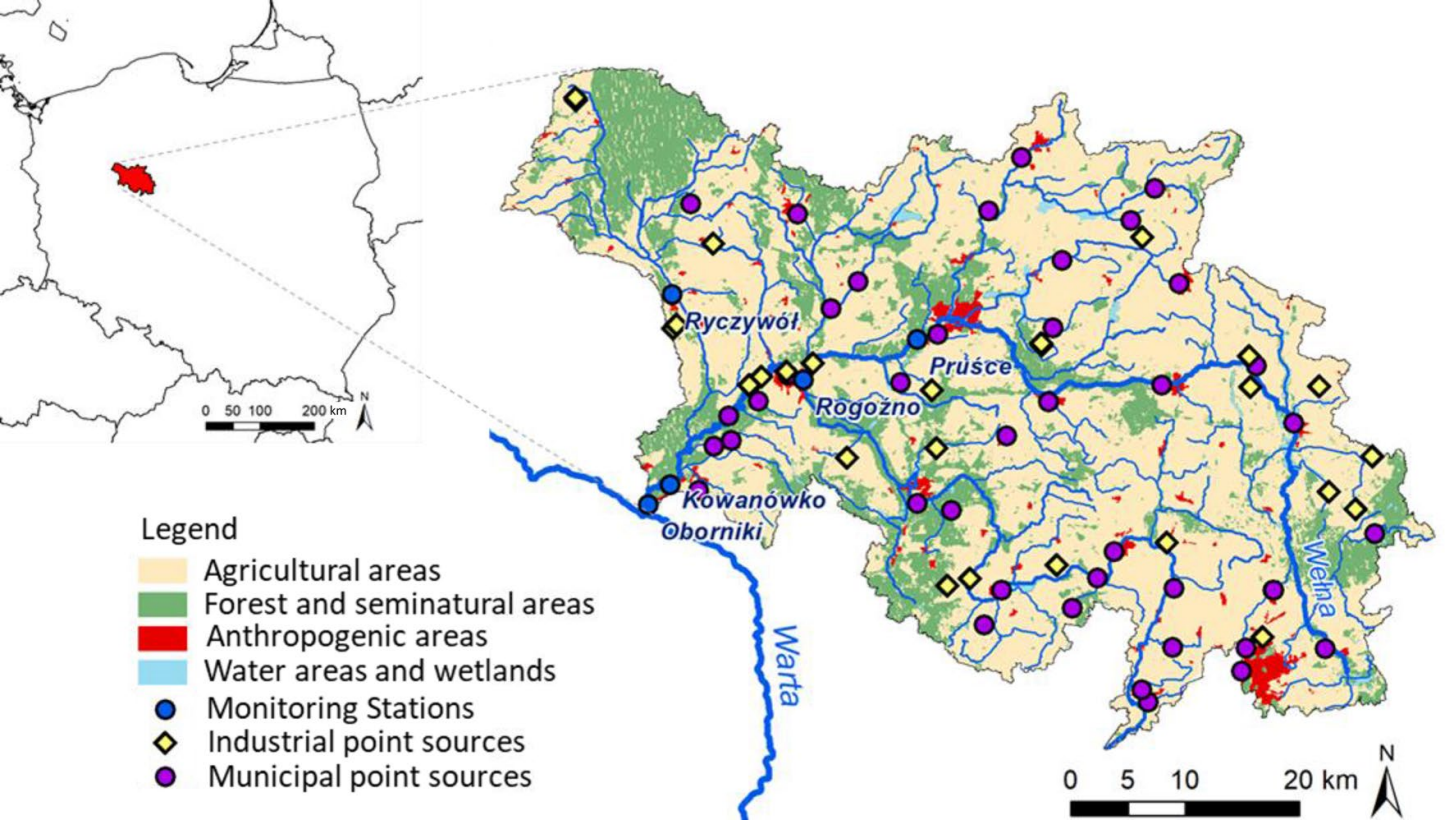
Localisation of the Wełna River catchment with its land use forms and nutrient sources. This figure was created using ArcGIS 10.2.1 for Desktop available at https://www.esri.com/en-us/home. Licence granted to Institute of Meteorology and Water Management.

The studied catchment (2 621 km^2^) is located in the central-western part of Poland, and constitutes a part of the Oder River basin. The Wełna River (118 km) discharges to the Warta River near the town of Oborniki^18^, with an average flow rate of 8.1 m^3^s^−1^ (1980–2019) in this profile^19^. The natural conditions in this catchment favour the development of intensive agriculture, which covers almost 72% of this area (1888 km^2^) and contributes to the high consumption of mineral fertilizers^20^. Forest areas cover another 22% of this catchment (589 km^2^), while urbanised ones only 4% (93 km^2^) (Fig. 1). The Wełna River catchment is inhabited by approx. 230,000 people, of which only approx. 74% is served by wastewater treatment facilities^21^.

### Input data

**Figure 2:**
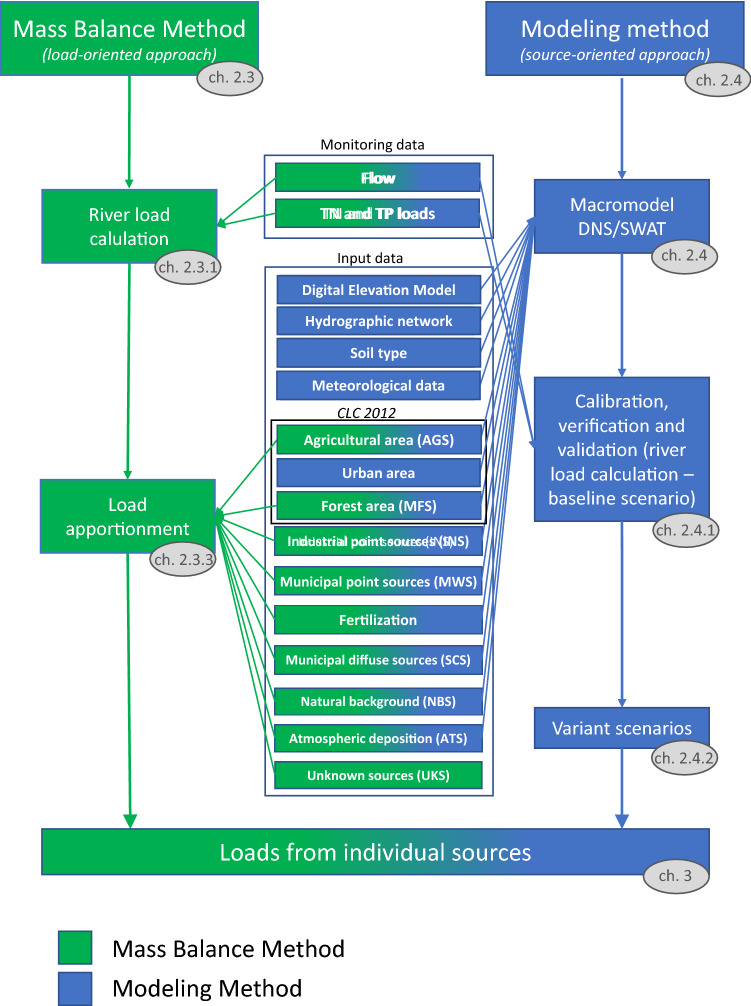
Methodology diagram with relevant chapters marked in grey ovals (green—steps and data used for Mass Balance method, blue—steps data used for Modelling method, green/blue—steps and data used for both methods).

Both the mass-balance method and the modelling method require a similar amount and type of input data (Supplementary Table S1). Basic information on the Wełna River daily flow rates and nutrient concentrations in the closing profile of the catchment (Oborniki) has been obtained from the state monitoring services (Institute of Meteorology and Water Management—National Research Institute—IMGW-PIB^13^ and State Environmental Monitoring^22^—SEM) (Supplementary Table S1). They have formed the basis for the estimation of the share of individual sources in the mass-balance method, as well as for the calibration of the Macromodel DNS/SWAT in the modelling method. Other data, such as maps of elevation, river network and soil maps, as well as meteorological data, necessary for the development of an accurate representation of the studied catchment area on the Macromodel DNS/SWAT digital platform, were also obtained from state repositories. Data on the land use comes from the Corine Land Cover^8^, while detailed information on nutrient sources has been obtained mostly from the Local Data Bank of statistical information. The utilisation of the collected database has been presented in Fig. 2, and described in the following text. The comparison of the results for nutrient loads from both method was based on the year 2017, which was characterised by the maximum amount of monitoring data for both flows (365 measurements) (IMGW-PIB) and total nitrogen (TN) and total phosphorus (TP) (12 measurements–SEM). The average air temperature in 2017 in Poland was 1.5 °C higher than the long-term average (1971–2000) and was over 10 °C which resulted from the warm autumn and the end of the year. The time of the snow cover presence was shorter than the long-term data, and the rest of the year was classified as thermally normal.

In terms of precipitation, 2017 was assessed as wet, similarly due to rainy autumn and summer. In the Wełna River catchment area, the annual rainfall was about 770 mm, however high variability of precipitation conditions in particular months, from extremely wet to very dry, should be noted^23^. Therefore hydrologically, 2017 was considered normal with the flows only slightly lower than the long-term average.

### Mass-balance method

The first method used for the quantification of sources and loads in the studied area was the static mass-balance method. It is widely used by the Polish administration authorities responsible for water management^17^. This method is based on the assumption that the sum of the nutrient loads in the river’s closing profile (selected based on access to the monitoring data) and its retention in the catchment equals the emission of nutrients in a given time. Such assumption allows the apportioning of the river loads among identified sources and the estimation of their contribution to the total loads, based on known or assumed values of their retention.

#### River load calculation

The total load of nutrients discharged from the catchment was calculated using the daily flow rate and nutrient concentrations in the closing profile of the catchment area (Oborniki, Fig. 1) from the SEM (Supplementary Table S1). The daily river load was calculated using the following Eq. ^5^:

1 $${L}_{river}=0.0864\sum_{t=1}^{n=365}{({Q}_{t}\cdot {C}_{t})}_{t}$$

where: *L*_*river*_ is the annual load [kga^-1^], *n* is the number of days, *t* is the consecutive day, *C*_*t*_ is the concentration [mg L^-1^], *Q*_*t*_ is the mean daily flow rate [Ls^-1^], and 0.0864 is the unit conversion.

Due to the fact that the flow rate is measured daily and nutrient concentrations only 12 times a year, the linear interpolation method^5^ was used to obtain the daily concentration values:

2 $${x}_{k}={x}_{a}+k\cdot \frac{{x}_{b}-{x}_{a}}{n+1}$$

where: *x*_*k*_ is the interpolated concentration value, *x*_*a*_ is the first of the two measured concentration values between which the concentrations are interpolated, *x*_*b*_ is the second of the two measured concentration values between which the concentrations are interpolated, *k* is the consecutive number of missing value and n is number of missing values.

#### Source apportionment

For the mass-balance method, data on nutrient loads for source apportionment (emission inventory) was collected in order to proceed with further calculations. The calculations were performed for 2017, due to the availability of river monitoring data and the nutrient sources were divided into 7 categories, based on the HELCOM guidelines^5^: municipal (MWS) and industrial (INS) point sources, municipal diffuse sources (SCS), forestry (MFS), agriculture (AGS), natural background (NBS) and atmospheric deposition (ATS). The category of “unknown sources” (UKS) was taken into account, in order to include possible discrepancies in nutrients load apportionment, and to cover eventual differences between calculated river load and inventoried emission.

The MWS loads were calculated on the basis of the number of inhabitants served by the wastewater treatment plants (WWTPs)^21^. In the Wełna River catchment, 151 771 inhabitants were served by the 12 WWTPs covered by the National Wastewater Treatment Program (NWTP)^24^, which provides information on the total discharge volume from each facility. For 5 of these plants, information on influent/effluent nutrient concentrations was also available, allowing the direct calculation of discharged loads. For the remaining seven facilities, the loads were calculated on the basis of the mean influent concentration information, available for the WWTPs covered by the NWTP (80 mgL^−1^ and 12 mgL^−1^ for TN and TP, respectively), and approximated nutrient reduction level in non-biological WWTPs. This reduction level, based on data from the NWTP, was set at 65% for TN and 35% for TP^24^. Another 19 350 inhabitants of this catchment were connected to the small WWTPs, not included in the NWTP. This part of the MWS load was calculated using the mean daily wastewater outflow (0.12 m^3^day^−1^ per person), the same mean nutrient concentrations and reduction levels as presented above. Additionally, the remaining 25% of the catchment’s population (58,000) is not connected to any WWTP and uses septic tanks and other types of individual wastewater treatment systems. The load from this source was expressed as SCS, and calculated using unit loads set on 11 gday^−1^ per person and 1.6 gday^-1^ per person for TN and TP, respectively^17^. The industrial nutrient input information (INS) was gathered directly from the Statistics Poland office database^21^.

The AGS load was calculated using nitrate and phosphate concentrations in shallow groundwater (90 cm below the ground surface), from 22 sampling points located on agricultural areas in the Wełna River catchment^17^. Concentrations were recalculated to TN and TP respectively and averaged. Thus, the obtained mean concentrations were 8.25 mgL^−1^ of TN and 1.92 mgL^−1^ of TP. Subsequently, load values were calculated by multiplying these concentrations by the outflow from agricultural areas, calculated as a share of the total catchment outflow respective to the agricultural use of the area. The calculated loads were multiplied by coefficients reflecting the share of monitored outflow (groundwaters and tile drainage) from the agricultural runoff (1.11 for TN and 4.17 for TP)^25^. Subsequently, the natural background (NBS) was subtracted from the AGS load.

The load corresponding to NBS was calculated using the total catchment outflow and nutrient concentration values reflecting conditions in undisturbed areas of pre-human activity, set as 0.15 mgL^−1^ and 0.02 mgL^−1^, for TN and TP respectively^17^. The MFS load was also calculated in a similar way, using nutrient concentrations set to represent forest catchment as 0.31 mgL^−1^ and 0.038 mgL^−1^^17^ and the outflow calculated as the share of the total catchment area, respective for the catchment part covered by forest. Also in this case, the NBS load has been subtracted. As for the ATS load, data on pollutant deposition into the ground from precipitation was taken from the SEM network^26^. This data was based on precipitation and its chemistry measurements taken from 22 monitoring stations covering the entire territory of Poland. The total load from the point and diffuse sources was calculated by adding the loads mentioned above. The eventual difference between the river load ("River load calculation" Section) and inventoried emission ("Mass-balance method" Section) accounted for the other sources (UKS).

#### Load apportionment

The contribution of each source to the calculated river load was calculated based on a simplified equation modified from HELCOM^5^:

3 $${L}_{river}=DP+LOD-RP-RD$$

where: L_river_ is the river load [kga^−1^], DP is the load from point sources (MWS and INS) [kga^−1^], LOD is the load from diffuse sources (SCS, ATS, MFS, AGS and, NBS) [kga^−1^], RP is the point source retention [kga^−1^] and RD is the diffuse source retention [kga^−1^].

In the adopted mass-balance method, it is assumed that nutrient load from the point sources (DP) is introduced directly into the river bed phase, while load from the diffuse sources (LOD) is discharged into both phases of the catchment, land and river bed ones. In both phases, self-purification processes are taking place, resulting in the reduction of nutrient loads on the way from the source to the catchment closing profile. However, due to the limited amount of data, the self-purification processes in the river have been omitted, therefore the point source retention (RP) equalled 0 kga^−1^. Subsequently, the diffuse source retention (RD) has been estimated on the basis of the difference between each nutrient load of the river (L_river_) and the point sources (LOD). The remaining river load has been then attributed proportionally to the contribution of the particular diffuse sources to the total source apportionment (emission inventory).

### Modelling method

The digital platform, the Macromodel DNS with the SWAT module^27–32^, was used for comparison for the nutrient balancing method described in "Mass-balance method" Section. This advanced dynamic tool tracks nitrogen and phosphorus migration paths in the river basin taking into account their spatial and temporal variability. For this purpose, it takes into account a very extensive input database, similar to that used in the mass balance method (Supplementary Table S1). Natural and anthropogenic processes that affect the transport and transformation of nutrients, are also part of this platform. The SWAT module (version 2012) is a tool which operates in the spatial information system (GIS) and is fully integrated with it. Using the digital elevation model (DEM), the SWAT module divided the entire analysed Wełna River catchment into a total of 225 sub-catchments of an average area of 11.5 km^2^. The subsequent use of data on land use (forests, agriculture and urbanised areas) and the types of soils (31 classes) allowed the authors to identify a total of 2824 hydrological response units (HRUs), homogeneous in terms of vegetation, soil and topography^33^. Afterwards, a simulation of soil water content, evapotranspiration, surface runoff, primary and underground flows was carried out in accordance with the water balance Eq. (4), which represents the basis for the quantitative component and the HRU.

4 $${SW}_{t}={SW}_{0}+\sum_{i=1}^{t}({R}_{day}-{Q}_{surf}-{E}_{a}-{W}_{seep}-{Q}_{gw})$$

where: *SW*_*t*_ is the final soil water content (mm H_2_O), *SW*_*0*_ is the initial soil water content (mm H_2_O), *R*_*day*_ is the amount of precipitation (mm H_2_O), *Q*_*surf*_ is amount of surface runoff (mm H_2_O), E_a_ is the amount of evapotranspiration (mm H_2_O), *W*_*seep*_ is the amount of water entering the vadose zone from the soil profile (mm H_2_O), *Q*_*gw*_ is the amount of return flow (mm H_2_O).

The model directs all runoff flows generated by each HRU through the channel network, thus simulating a catchment. The water balance equation also represents a basis for the simulation, transport and transformation of nutrients required for the quantitative component of the model. This tool models forms of nitrogen, organic and inorganic , different forms of phosphorus in soil^34^, as well as organic nitrogen and phosphorus forms associated with plant residues, microbial biomass and soil humus^35–38^. Final results of simulations are produced by the SWAT model as all the forms of nitrogen and phosphorus (in kilograms of N and P per a time unit, respectively) are then summed up to give TN and TP values. To verify that the model properly predicts TN and TP values its results are calibrated with the TN and TP values resulting from SEM, as described in Sect. 2.4.1. Moreover, the particular forms of nitrogen and phosphorus have also been compared with the modelling results (Supplementary Table S4). A detailed overview of the migration and transformation pathways of nitrogen and phosphorus forms in the catchment has been presented in the Supplementary Information (Sect. S1), while mathematical description of these processes is included in the theoretical documentation of the SWAT model^39^.

Similarly, as in the case of the mass-balance method, diffuse sources of nutrients from agriculture (AGS), forestry (MFS) or urban areas (URB) in SWAT were simulated in the land phase of the catchment. In the land phase, the model simulates both the infiltration of nutrients into the soil (fertilization, plant biomass, precipitation) and their removal from it (volatilization, denitrification, erosion, surface runoff). Additionally, changes in the distribution of nutrients in the soil (uptake by plants) and the low mobility of phosphorus itself are also taken into account^39–41^.

Pollutants from municipal and industrial point sources (MWS, INS) are introduced directly into the river bed phase. The exception here is the nutrient load from municipal diffuse sources (SCS) which, reduced as a result of the self-purification processes taking place in the land phase, is also treated in the model as point sources. The SCS nutrient load mainly derives from leaking or illegally emptied septic tanks. For this purpose, septic tanks have been divided into three types: leaky, partially illegally emptied, and sealed septic tanks, legally emptied. The loads from the legally emptied tanks are included in the effluents from WWTPs reported in the catchment. While for the remaining types, their loads are calculated using factors depending on their effectiveness in nutrient removal (15 – 50%). The final nutrient load derived from these types of facilities is then calculated, taking into consideration the number of inhabitants using the different types of septic tanks and the average chemical composition of wastewater^21^.

The load of nutrients from the atmospheric deposition (ATS) affects both land and river phases due to the presence of two deposition mechanisms in the SWAT module, i.e., wet and dry deposition. The model also allows for the determination of nutrient loads generated as a result of natural processes of nitrogen and phosphorus transformation and transport in the soil, with the omission of all anthropogenic pressure—natural background (NBS).

#### Calibration, verification and validation

The SWAT module for the Wełna River has been calibrated, verified and validated using the SWAT-CUP software^42^. For the quantitative component (water circulation in the catchment), the implemented daily flow data (source: IMGW-PIB) for the period of 18 years (2001–2018) came from the water gauge stations on the Wełna River (Pruśce and Kowanówko) and its tributary (the Flinta River-Ryczywół) (Fig. 1). The qualitative component (nitrogen and phosphorus concentration in the catchment) was gathered from the SEM stations localised at the Wełna River (Oborniki and Rogoźno) (Fig. 1) and covered a period of 13 years (2005–2018). Three statistical measures, coefficient of determination (R2)^43^, percent bias (PBIAS)^44^, and Kling-Gupta efficiency (KGE)^45^, have been used to indicate the Wełna River model performance (Supplementary Tables S2 and S3). In terms of the quantitative component, the calibration and verification coefficients R2, KGE and PBIAS classified the model performance generally as good and very good for the main river (Wełna), and satisfactory and good for its tributary (Flinta). During the validation procedure, all coefficient values rated the model performance for daily flow simulations as very good. In terms of qualitative components, the model performance for TN simulations can be considered as very good or good, according to the all-applied coefficients. Lower model performance, mostly satisfactory, was observed for TP mainly due to the variability of phosphorus temporal distribution patterns (Supplementary Table S2). The entire process was described in detail in Orlińska-Woźniak et al^46^.

#### Variant scenarios

**Figure 3:**
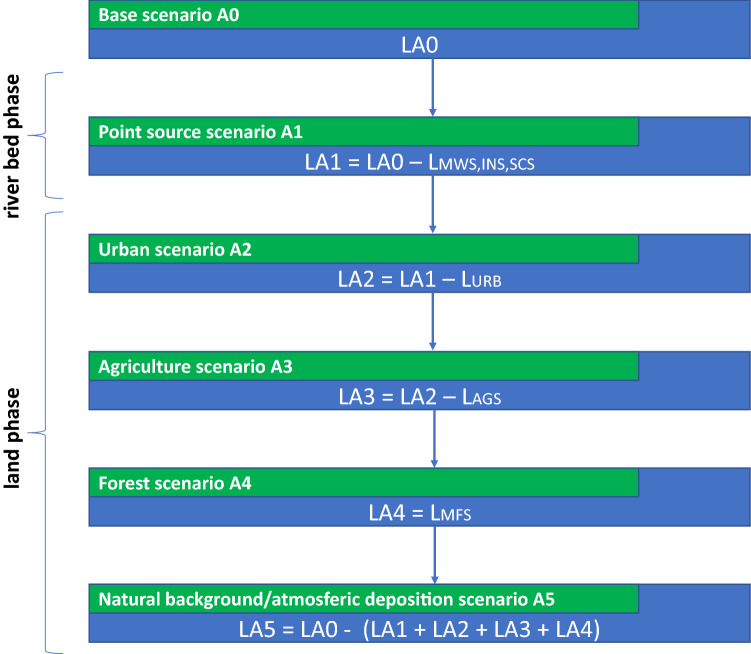
Variant analysis diagram for assessment of nutrient loads (L) for particular modelling scenarios and sources described in the text: MWS, INS, SCS—point sources, URB—urban, AGS—agricultural, MFS—forest.

In order to determine the contribution of individual sources to the total load of nutrients in the profile closing the analysed catchment, a final simulation of the model was used and subjected to calibration, verification and validation procedures, and called the baseline scenario (A0). Subsequent so-called variant scenarios (A1–A5), i.e. model simulations, were developed. In variant scenarios the values of selected parameters were changed in relation to the A0 scenario. This was used both in the river bed phase for point sources (A1) and for individual diffuse sources (A2–5), thus imitating surface changes for particular types of land use in the land phase of the catchment (Fig. 3).

In the A1 scenario, all parameterized and aggregated point sources (MWS, INS, SCS) for each relevant sub-basin (L_MWS,INS,SCS_), were removed from the model to calculate their contribution to the total nutrient loads in the closing profile of the studied catchment (LA1).

In the next two scenarios (A2 and A3), concerning urban and agricultural land use, their surface areas (5 663 ha and 192 917 ha, respectively) were successively replaced by the forest land use. This procedure was based on the assumption that the forest is the primary type of land use for this catchment area^47^. In order to completely eliminate the influence of these areas, the nutrient loads from the relevant surface area occupied by forest land use were subtracted, in order to estimate the contribution of urban and agricultural land (L_URB_ and L_AGS_, respectively).

The change in land use from urbanised and agricultural areas to forest areas increased their percentage of the catchment area to almost 100%, thus the original image of the catchment area and the nutrient load at its mouth. On this basis, in scenario A4, the nutrient load from forests L_MFS_, which currently occupy only 20% of the catchment area (A0), flowing to the closing profile, was calculated from the proportion.

The A5 scenario is the difference between the nutrient load from the A0 scenario and the sum of the remaining loads from the subsequent variant scenarios (A1–A4). In this way, both the natural background (NBS) and atmospheric deposition (ATS) were taken into account.

## Results

For the purposes of the current research, two significantly different methods of source identification and estimation of their contribution to the total nutrient load have been selected. The confrontation of the mass-balance method with the modelling approach revealed clear differences in the total estimates for the closing profile of the analysed catchment (Oborniki) and also in the individual contribution of each source. As for the river load for this profile, used as a basis for the mass-balance method, calculated using data from the SEM and flow measurements, the loads amounted to approx. 3 127 ty^−1^ (tonnes per year) and 63 ty^−1^ for TN and TP, respectively. While the modelling results for the baseline scenario (A0) demonstrated values lower by approx. 943 ty^−1^ and 11.3 ty^−1^ for TN and TP, respectively (Table 1).

**Table 1:** Nutrient loads and source contributions for the mass-balance and modelling sources methods at the calculation profile of Oborniki (2017).

Source:		Mass-Balance method				Modelling method				
		TN load [kgy^-1^]	%	TP load [kgy^-1^]	%	Scenario	TN load [kgy^-1^]	%	TP load [kgy^-1^]	%
Municipal point sources	MWS	132 827	4.25	21 530	34.17	A1	192 898	8.8	13 411	25,9
Municipal diffuse sources	SCS	234 829	7.51	496	0.79					
Industrial point sources	INS	1 045	0.03	69	0.11					
Urban	URB	–	–	–	–	A2	54 107	2.4	2 428	4,8
Agriculture	AGS	2 474 435	79.1	32 048	50.86	A3	1 795 696	82.2	28 120	54,4
Forestry	MFS	13 800	0.44	23	0.04	A4	53 871	2.5	844	1,6
Natural background	NBS	57 522	1.84	7 670	12.17	A5	88 595	4.1	6 899	13,3
Atmospheric deposition	ATS	71 644	2.29	1 178	1.87					
Unknown sources	UKS	141 815	4.53	0	0.00	–	–	–	–	–
SEM		3 127 916	100	63 013	100	A0	2 185 167	100	51 701	100

The contribution of particular nutrient sources has been determined depending on the capabilities of both methods and limitations of the available data. Nutrient loads discharged from the different types of wastewater treatment facilities used by the inhabitants of the catchment (MWS, SCS), as well as industrial point sources (INS), have been calculated separately in the mass-balance method. While in the modelling approach, all three sources have been aggregated into one scenario (A1). The difference between these sources in both methods is approx. 56%, with the loads much higher for the mass-balance method, especially for TN from the diffuse sources (SCS) due to the different retention percentage calculated for each nutrient using the mass-balance method.

In terms of diffuse sources reflecting different types of land use in the studied catchment, the urban areas (URB) have not been included in the mass-balance method due to the lack of suitable data. Whereas in the modelling method, this source accounted for 2.4% and 4.9% of the total TN and TP loads (A2), respectively. As for the agricultural activity (AGS) in the Wełna catchment, both methods indicated this source of nutrients as a main pressure in this area. Despite the substantial difference in TN load between both mass-balance and modelling approaches (approx. 767 ty ^− 1^), the contribution of this source to the total load was consistent, reaching 82% for each method. As for TP, this contribution accounted for 61% and 54% for the mass-balance and the modelling method (A3), respectively. As for the forest areas (MFS) nutrient loads were considerably higher by approx. 40.1 ty^−1^ and 0.7 ty^−1^ for TN and TP, respectively, in the modelling approach (A4).

The natural background (NBS) and atmospheric deposition (ATS) nutrient pressures were calculated separately in the mass-balance method, while the adopted modelling approach required their aggregation into one scenario (A5). The comparison of these loads between both methods revealed higher loads of TN by approx. 35.9 ty^−1^ for the mass-balance scenario, while higher load of TP by approx. 5.3 ty^−1^ was observed for the modelling method. As for the other sources (UKS), resulting from the differences observed in the mass-balance method between the river load and inventoried emissions, their contribution accounted for 2.4% of the TN total catchment load. In the modelling method, however, this source has been omitted since the presented list of scenarios already included all types of source data originally used to build the SWAT model for the Wełna River.

## Discussion

The comparison between the mass-balance method based solely on load-oriented approach and results obtained from the Makromodel DNS/SWAT for the medium-sized catchment, which has been presented in this study, has demonstrated discrepancies in the total nutrient loads and in the contribution of individual sources.

**Figure 4:**
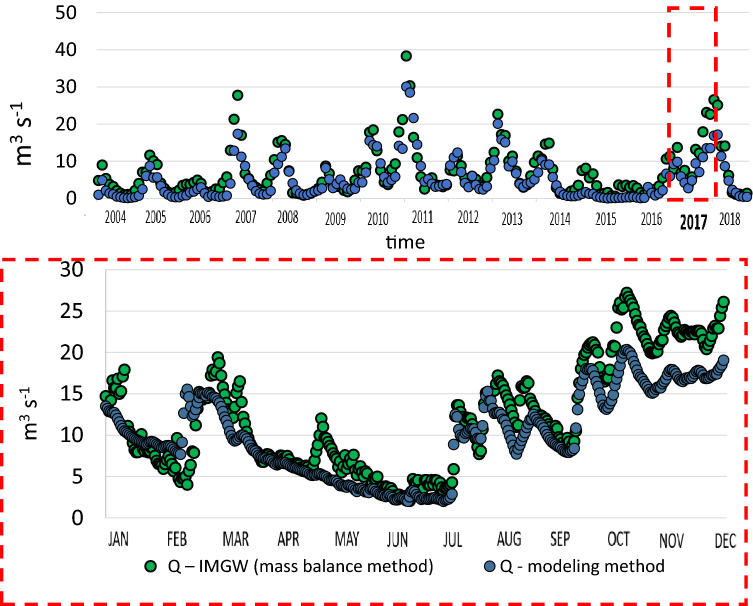
Matching of simulation results with monitoring data for multi-year flows (2017 featured in a dashed box).

The river flow measurements and their translation into the model can be identified as the first source of these discrepancies. For the purposes of this study, the flow rate monitoring data (2017) has been taken from the Kowanówko gauge station (IMGW-PIB), where the water level is measured continuously (Fig. 4). The flow data from the very same profile, and from two others (Pruśce and Ryczywół) has also been used to calibrate, verify and validate the Wełna River model for the period of 2001–2018, which covers the entire flow cycle (low, medium and high river flows). Despite the very good or good model performance with regard to river flow monitoring data (Supplementary Tables S2 and S3), generally an underestimation of the peak flows could be observed (Fig. 4). This phenomenon has been reported quite frequently^48,49^ and related to the curve number method used in the model adopted to estimate direct runoffs from rainfall events^50,51^.

**Figure 5:**
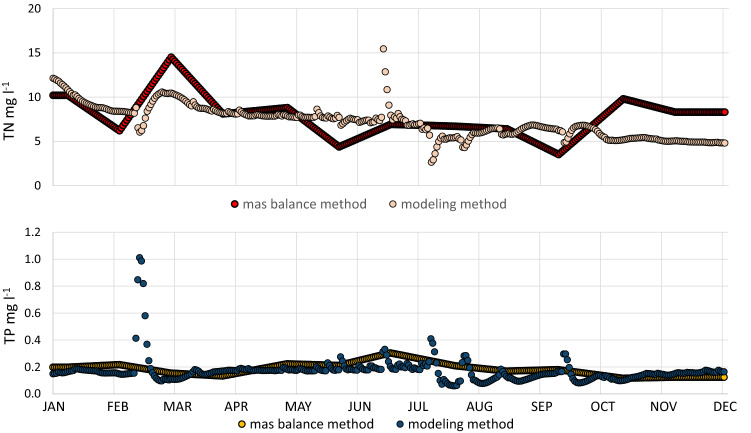
Comparison of simulation results and interpolated monitoring data for nitrogen and phosphorus concentrations for the Wełna River (Oborniki profile, 2017).

To calculate total loads in the mass-balance method (Eq. 1) TN and TP concentrations obtained from the SEM at the Oborniki monitoring station (2017), localised approx. 5 km downstream from the gauge station (Kowanówko), have been used. This difference has been neglected since no other tributary discharges into the main river at this distance. Since the quality monitoring in 2017 in Oborniki was performed with a monthly frequency, the linear interpolation method (Eq. 2) was used to obtain the daily concentration values (Fig. 5). To confront these values with nutrient concentrations from the modelling approach, the TN and TP values were calculated from the simulated loads. This comparison (Fig. 5). clearly shows that daily variability of nutrient concentrations is high and not considered in the mass-balance method. The uncertainty of measurements performed under the SEM system were estimated at 19% and 16% for TN and TP concentrations, respectively. Taking into consideration that the uncertainty of flow measurements has been estimated at 5%, the total uncertainty for mass-balance loads could be estimated (based on the root mean square error propagation method) at 19.6% and 16.7% for TN and TP loads, respectively. Whereas the uncertainty of nutrient loads in the modelling approach has been estimated at 12.5% and 20% for TN and TP loads, respectively.

As noted above, the proposed hybrid approach is beneficial in terms of both quantitative and qualitative assessments. Reliable flow values can be obtained for the ungauged parts of catchments which facilitates hydrological estimations. Moreover, combination of the static and dynamic methods also eliminates a common problem in Poland of the lack of consistency between location of hydrological (IMGW) and quality (SEM) monitoring points. Also, in the modelling step of the hybrid approach one can obtain flow values for almost any computational profile of the catchment, and subsequently pollutant loads, with much higher resolution of the obtained data. Therefore, instead of deficient linear interpolation of daily nutrient concentrations from SEM measurements (Fig. 5) the simulated results with any time resolution can be taken into consideration.

The distribution of total TN and TP loads among the particular sources (Table 1) depicts anthropogenic activity related to municipal and industrial wastewater production as the second highest source of nutrients. It contributes to 11.0 and 36.0% according to the mass-balance method (MWS, SCS, INS), and 8.8 and 25.9% according to the modelling approach (A1), for TN and TP respectively. Due to the mainly agricultural character of studied catchment, the contribution of industrial sources (INS) can be considered as marginal, while substantial nutrient loads are discharged from the wastewater treatment facilities. In both approaches, the discharges from different size WWTPs are regarded as point sources, releasing wastewater directly into the river bed phase.

However, the difference between the two methods is evident in this phase, since the static approach does not take into account the numerous natural self-cleaning processes. Thus, it is the next stage of nutrient balancing, the hybrid approach on the scale of individual catchments allows for filling these gaps using the functionality of the model, that includes simulation of processes such as transport, biodegradation, transformation, dilution, diffusion, deposition and accumulation of nutrients^28^. Consequently, one should expect much lower values of the modelled loads of nutrients on the discussed profile then in a simple mass-balance method.

**Figure 6:**
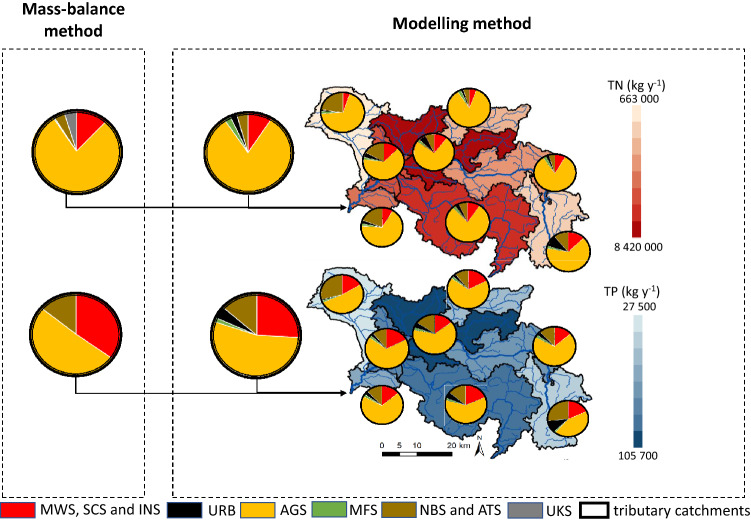
Contribution of nutrient sources in TN and TP loads under the mass-balance and modelling method. This figure was created using ArcGIS 10.2.1 for Desktop available at https://www.esri.com/en-us/home. Licence granted to Institute of Meteorology and Water Management.

The relatively low level of urbanisation in the analysed catchment, and hence the sparsity of households, impacts wastewater management in this area, which is still largely based on septic tanks^52,53^. The key factor for the calculation of the contribution of this source (SCS) to the total loads in both methods, is related to nutrient retention in the land phase. Significant differences between the adopted retention estimation methods lead to considerable differences between TN and TP load values. In the case of the mass-balance method, retention is calculated separately for nitrogen and phosphorus and based on the comparison between river loads and the sum of inventoried emissions in the catchment (source-oriented approach). Since in the studied catchment the nitrogen river load exceeded inventoried emissions, zero retention was assumed for this nutrient. As for phosphorus, the river load was much smaller than the inventoried emissions, therefore 99% of the retention was adopted and attributed solely to the diffuse sources, according to methodology described in 2.3.3. In the case of the modelling method, the retention of TN and TP resulting from anaerobic processes in septic tanks and transport of nutrients between land and river bed phases was included in the group of point sources. Moreover, in the case of sources where nutrient loads depend on the form of land use, the mass-balance method relied on an assessment based on outflow relative to the respective area of the catchment and TN and TP concentrations, measured or adopted for the particular land use form. In the modelling method, nutrient loads for these sources have been simulated based on land features embedded in the HRU system (slopes, soil type, land use) and combined with the meteorological and hydrological data. Eventually, the SWAT module simulates five different forms of nitrogen and six different forms of phosphorus, taking into account different pathways of nutrients delivery into the catchment and their removal through e.g. uptake by plants, volatilization, erosion, surface runoff, etc. It should be also noted that the HRU system allows the user to determine nutrient loads in any chosen spatial and temporal scale. Analyses based on HRU units, here aggregated to the Wełna River tributaries’ catchments (Fig. 6), display differences between different parts of the studied area, indicating changes in specific nutrient source contribution in 2017, and allowing to improve considerably information resolution.

As visible in Table 1 and Fig. 6 the urban areas (URB), which account for approx. 0.5% of the Wełna catchment, have been omitted in the mass-balance method, due to the lack of pertinent data on nutrient concentrations. In the modelling approach (A2), three categories of urban areas depending on the intensity of development (continuous, discontinuous, industrial) characterised by the different share of impervious surfaces have been taken into consideration. The resulting nutrient loads were relatively low, however the noticeably higher value for TP should be highlighted. Many published records indicate that indeed runoff from urban areas is rich in phosphorus compounds^54–57^ due to industrial activities, dust particles from roads and construction sites, burning of fossil fuels, fertilizers and biogenic particles from green areas.

**Figure 7:**
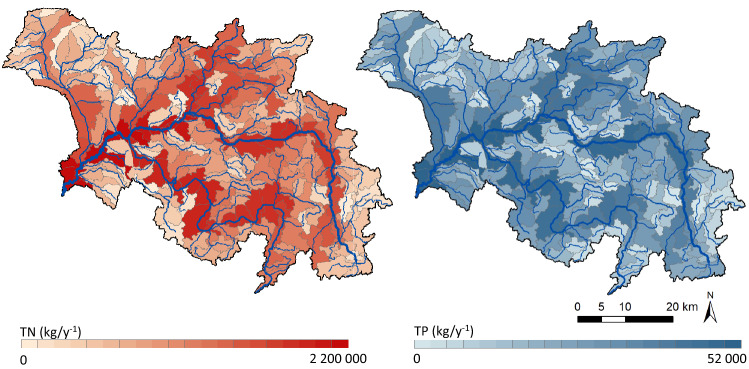
Distribution of TN and TP loads in individual sub-catchments of the Wełna River according to the modelling method. This figure was created using ArcGIS 10.2.1 for Desktop available at https://www.esri.com/en-us/home. Licence granted to Institute of Meteorology and Water Management.

Since approx. 75% of the total Wełna catchment area represents agricultural land use (AGS), this source plays the dominant role in both methods, contributing to approx. 82 and 54–61% of the TN and TP loads, respectively. Although relative contributions in both methods are at a similar level, the absolute loads are lower in the modelling method (A3) by 30% for both nutrients. To adapt the mass-balance method to the Wełna catchment conditions, the measured nitrate and phosphate concentrations in shallow groundwater in this area and specific coefficients have been used ("Source apportionment" Section). As for nutrient retention, zero nitrogen retention and 99% phosphorus retention has been implemented, as for other diffuse sources. The Macromodel DNS/SWAT, in turn, is a tool with a very extensive agricultural module^34,40^ and takes into account numerous data related to each type of the crop and agricultural practices (e.g., agrotechnical treatments, fertilization, crop rotation), therefore its results should be considered as more detailed. Moreover, spatial distribution of TN and TP loads in this dynamic approach can be presented in a manner allowing for clear identification of specific areas of concern (hot-spots) (Fig. 7). Additionally, when previously described^32^ a multi-stage delimitation procedure applied, the precise delineation of only those areas within the catchment, which actually contribute to the nutrient pollution and eutrophication, could be determined.

As for the remaining 20% of the land use covered by forestry (MFS), its contribution to TN and TP loads according to the mass-balance method was minimal, which seems to be an artefact of the adopted low concentrations representing this type of land use. However, the high ability of forest soils to retain and release nutrients through different biotic and abiotic mechanisms is also a well-known phenomenon^58,59^. The estimated loads (A4) according to the modelling approach were noticeably higher, although they still represented the lowest contribution to the nutrient content in relation to their surface area. Nutrient loads from the two remaining sources, natural background (NBS) and atmospheric deposition (ATS), were discharged both into the land and into the river bed phases. Although estimates according to both methods were based on a similar concept, the differences reached 30% and 80% for TN and TP, respectively. In the case of NBS, an attempt was made to recreate nutrient concentrations from the pre-human period, using coefficients on the natural background in undisturbed catchments^17^ according to the mass-balance method, and creating a variant scenario in a modelling approach where the entire catchment area was covered by forestry without any human activities^47,60^. As for ATS, which was combined in a single scenario along with NBS in the modelling simulations (A5), the data on pollutant atmospheric deposition was taken into consideration. However, phosphorus has not been included in the modelling method due to the limitations of the SWAT module, although both dry and wet deposition is minimal in Poland and does not exceed 0.34 kgPha^-1^y^-1^ (Supplementary Table S1).

As discerned above the hybrid approach is also beneficial when the land use aspect is taken into consideration. The modelling step allows for detailed analyses not only at the level of individual tributaries (Fig. 7), sub-catchment areas but also for the HRU units, which are defined, among others, based on land use. As a consequence, preparation and execution only dedicated remedial actions for specific areas of the catchment will be possible.

## Conclusion

Two different approaches have been used in parallel to balance nutrients in a medium-sized agricultural catchment. This comparison of two essentially different methods (mass-balance vs. modelling, static vs. dynamic) showed similar contributions for each nutrient source in absolute TN and TP loads, although the final estimates in the chosen calculation profile were divergent due to the various reasons related to the methods’ specificity. The mass-balance method, which is currently the primary method used by the public administration in Poland, offers a simple and relatively easy tool to balance nutrients at any given profile where monitoring data is available. However, this method heavily relies on numerous coefficients, while specific processes related to nutrient fate in water and soil environments are neglected. Moreover, the information resulting from this method is limited to the specific spatial and temporal coordinates (calculation profile and year). The modelling methods in turn allows for a detailed mapping of the nutrient load distribution which permits to point out specific areas where countermeasures should be undertaken. Moreover, temporal patterns (e.g., seasonal) of the nutrient loads could be easily tracked in a catchment of interest. Although, it should be noted that these advantages are achieved for the price of considerable effort related to the model building, and also its calibration, verification and validation procedures.

Since both methods offer substantial benefits we propose a hybrid approach to deal with nutrient problems both in a local context (defined catchment area) and in relation to nutrient budgeting for the whole country. In the first case, we suggest using a simpler and faster mass balance method to identify the problem at a specific place and time in a selected catchment area. The results obtained in this way will provide the basis for decision on whether to continue the research with the use of the modelling method. Which in turn will allow for determination of the nutrient load variability in time and space, and identification of hot-spots in individual sub-catchments. In the case of the hybrid approach in the context of the entire country (budgeting nutrients discharged from the entire territory of Poland to the Baltic Sea), we are suggesting to apply static and dynamic methods in parallel, since such gradually has been implemented in our country. Since, knowledge on nitrogen and phosphorus emissions and their impact on river loads in the Polish River Basin Management Plans are based mostly on static methods, further development of dynamic models is highly recommended. Development of models ultimately covering subsequent catchments according to presented methodology, consistent in a country wide scale, will result in a homogeneous approach to analyse nutrient pressures. In consequence, it will provide us with better information on the actions and measures needed to reach and maintain a good environmental state of water bodies. Moreover, simulation of these measures’ impact on nutrient reduction (scenario analysis) before their implementation would bring tangible benefits to Polish water administration and other stakeholders of water policy.

## Supplementary Information

Supplementary Information 1.
