# Supplementary material for: Estimation of nutrient loads with the use of mass-balance and modelling approaches on the Wełna River catchment example (central Poland)
Source: Sci Rep. 2022 Jul 29;12:13052. doi: 10.1038/s41598-022-17270-4 (PMC9338021; doi:10.1038/s41598-022-17270-4)
Supplement: Supplementary file 1 — Supplementary Information 1. [file 41598_2022_17270_MOESM1_ESM.docx]

**Estimation of nutrient loads with the use of mass-balance and modelling approaches on the Wełna River catchment example (central Poland)**

Authors: Damian Bojanowski^*1^, Paulina Orlińska-Woźniak^2^, Paweł Wilk^3^, Ewa Szalińska^4^

* corresponding author

1. AGH University of Science and Technology. Address: A. Mickiewicza Av. 30, 30-059 Krakow, Poland. e-mail: bojanows@agh.edu.pl^*^
2. Institute of Meteorology and Water Management - National Research Institute. Address: Podleśna 61, 01-673 Warsaw, Poland. e-mail: paulina.wozniak@imgw.pl
3. Institute of Meteorology and Water Management - National Research Institute. Address: Podleśna 61, 01-673 Warsaw, Poland. e-mail: pawel.wilk@imgw.pl
4. AGH University of Science and Technology. Address: A. Mickiewicza Av. 30, 30-059 Krakow, Poland. e-mail: eszalinska@agh.edu.pl

**Supplementary Information**

Section S1. The migration and transformation pathways of nitrogen and phosphorus forms in the catchment included in the SWAT module.

The SWAT model takes into account the organic and mineral forms of nitrogen and phosphorus in soils. Nitrogen and phosphorus enter the soil through fertilization with artificial fertilizers or manure, through plant biomass residues, binding by bacteria and precipitation. Phosphorus is removed from the soil by plant uptake and erosion, and nitrogen additionally by leaching, gaseous release and denitrification. Fig. 1 shows the main components of the nitrogen and phosphorus cycle in the SWAT model.


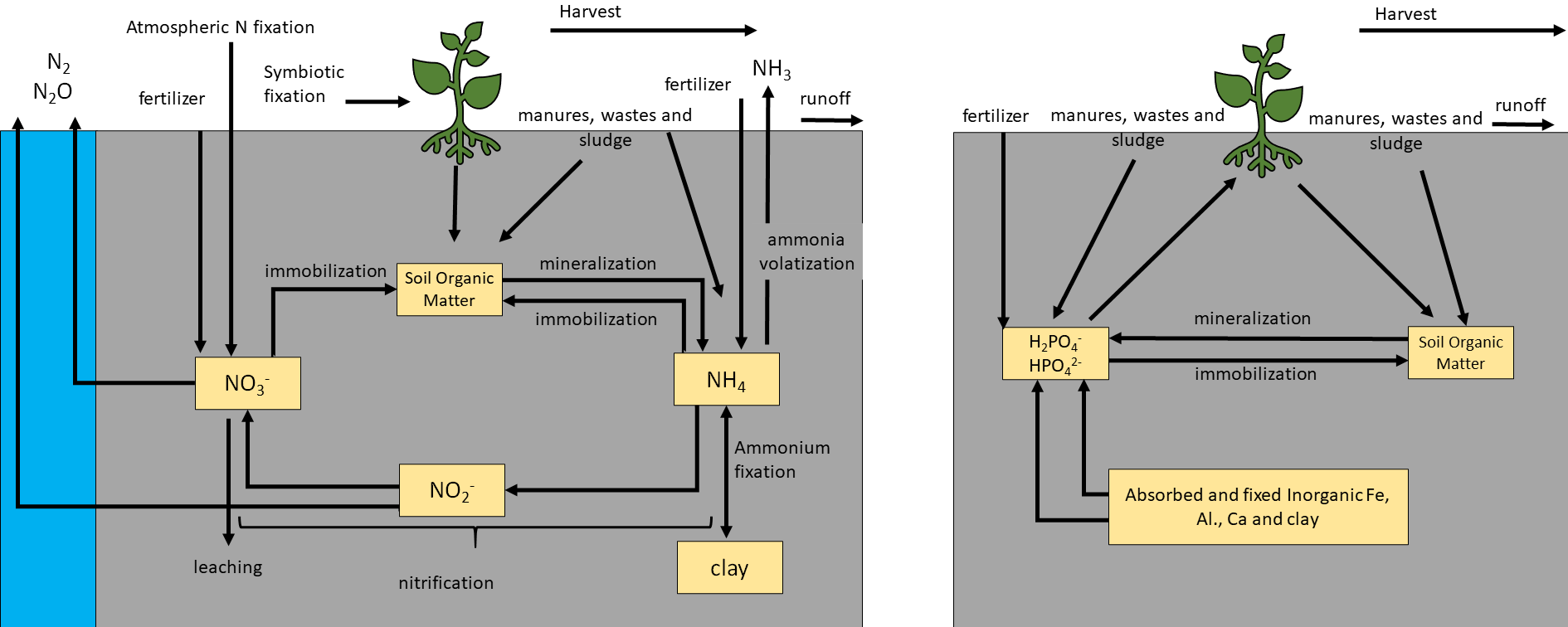


Fig. S1 The nitrogen and phosphorous cycles in SWAT. Altered from Neitsch, S.L. et al. 2011^[1][2]^. This figure was created using Microsoft Power Point version 2108 available at https://www.office.com/. License granted to AGH University of Science and Technology.

Fresh organic nitrogen and phosphorus are associated with plant residues and microbial biomass, while active and stable organic resources of these elements are associated with soil humus. Nitrogen and organic phosphorus associated with humus is divided into two forms, allowing for the variation in the availability of humic substances in the mineralization process.

The SWAT model describes the movement and transformations of nitrogen and phosphorus forms in the catchment area and models the different soil processes shown in Figures 2 and 3. Area changes in the distribution of nutrients in the soil as well as the nutrient use by plants are taken into account. In the model, it also allows to simulate the uptake of water and nutrients in the root zone, as well as transpiration and biomass production.


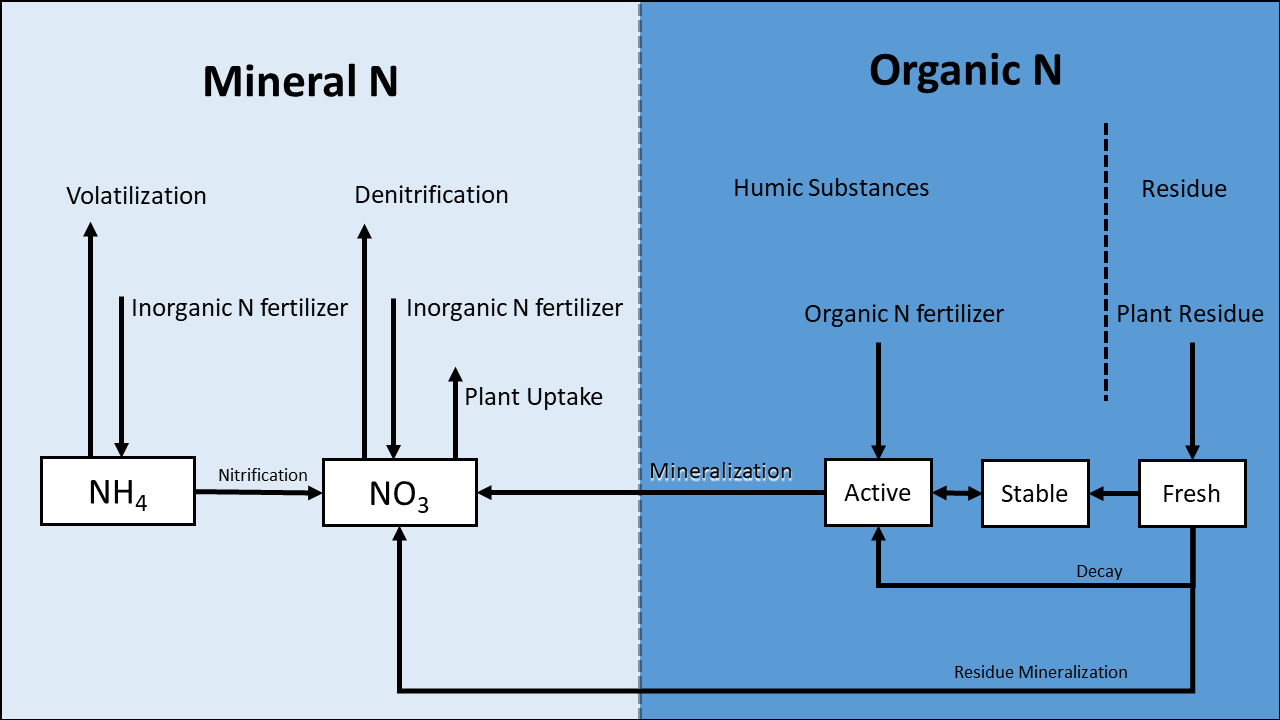


Fig. S2 Forms of nitrogen in soil described in the SWAT model along with transformation processes. Altered from Neitsch, S.L. et al. 2011^[1]^. This figure was created using Microsoft Power Point version 2108 available at https://www.office.com/. License granted to AGH University of Science and Technology.


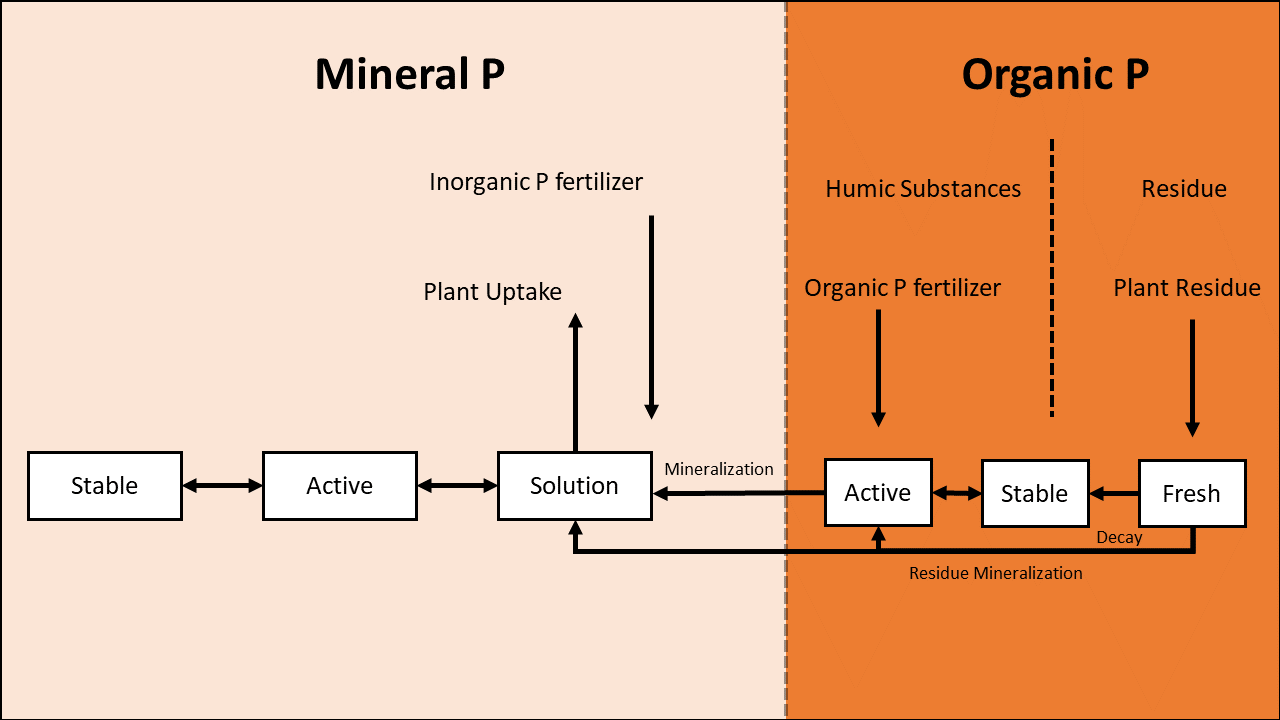


Fig. S3 Forms of phosphorus in soil described in the SWAT model along with transformation processes. Altered from Neitsch, S.L. et al. 2011^[1]^. This figure was created using Microsoft Power Point version 2108 available at https://www.office.com/. License granted to AGH University of Science and Technology.

The demand for the daily supply of nitrogen for plants is calculated as the difference between the actual concentration of a given element in the plant and its optimal concentration, which changes with the plant's growth. Additionally, the SWAT model uses nitrates and organic nitrogen, which can be removed from the soil by the movement of water. The amount of nitrates and organic nitrogen contained in surface and subsurface runoff and transported in the percolation process is calculated as the product of the water volume and the average concentration of nitrates in the analyzed soil layer. The movement of organic nitrogen with sedimentation is calculated using the charge function. As in the case of nitrogen, the plant uses phosphorus estimated using the approximate requirements of the daily phosphorus supply, calculated as the difference between the actual concentration of a given element in the plant and its optimal concentration. In addition to being used by plants, dissolved phosphorus in water and organic phosphorus can be removed from the soil by flowing water through it. Phosphorus is not a very mobile nutrient, therefore the interaction between surface runoff and dissolved phosphorus in the top 10 mm of the soil will not be complete. The amount of dissolved phosphorus removed in the runoff is predicted using an estimate of the phosphorus concentration in the top 10 mm of soil, runoff volume and the so-called runoff partition coefficient.

Table S1 Input data for both methodologies.

| Description | | | **Mass Balance Method** | **Modeling Method** |
| --- | --- | --- | --- | --- |
|  |  |  | **Data source and resolution** | |
| monitoring data | Flow | | [IMGW-PIB. Daily flow data from 2017 for the Kowanówko calculation profile. https://danepubliczne.imgw.pl/](https://danepubliczne.imgw.pl/) | [IMGW-PIB. Daily flow data from the period of 18 years (2001-2018) for the calculation profiles of Pruśce and Kowanówko (Wełna) and Ryczywół (Flinta). https://danepubliczne.imgw.pl/](https://danepubliczne.imgw.pl/) |
|  | TN, TP | | [Chief Inspectorate of Environmental Protection: State Environmental Monitoring https://www.gios.gov.pl/pl/stan-srodowiska/monitoring-wod](https://www.gios.gov.pl/pl/stan-srodowiska/monitoring-wod) | [Chief Inspectorate of Environmental Protection: State Environmental Monitoring. TN and TP data from the period of 15 years (2001-2015) for the calculation profiles Oborniki and Rogoźno. https://www.gios.gov.pl/pl/stan-srodowiska/monitoring-wod](https://www.gios.gov.pl/pl/stan-srodowiska/monitoring-wod) |
| input data | Digital elevation model DEM | | - | Nation Protection IT System.-10m, 1:20000. https://isok.gov.pl/index.html |
|  | Hydrographic network | | [State Water Holding-Polish Waters: Map of the Hydrographic Division of Poland. https://isok.gov.pl/hydroportal.html](https://isok.gov.pl/hydroportal.html) | |
|  | Soil type | | State Water Holding - Polish Waters: "Identification of pressures in water regions and river basins districts. Part II: Development of database of anthropogenic pressures. | Soil-agricultural map, 1: 100 000, 2.5m - Institute of Soil Science and Plant Cultivation and a map of forest soils in the scale of 1: 10 000, 2.5m - Polish State Forests (31 soil classes) |
|  | Meteorological data | | - | [IMGW-PIB - air temperature, precipitation, humidity, wind speed and total radiation, for 250 stations located directly in the basin, and within 20 km from its borders. https://danepubliczne.imgw.pl/](https://danepubliczne.imgw.pl/) |
|  | Land use | Agricultural area (AGS) | EEA: Corine Land Cover (CLC 2018). https://land.copernicus.eu/pan-european/corine-land-cover | [Corine Land Cover (CLC 2012), Landsat 8 satellite images http://clc.gios.gov.pl/index.php/26-clc-2012, https://bdl.stat.gov.pl/BDL](https://bdl.stat.gov.pl/BDL) |
|  |  | Urban area |  |  |
|  |  | Forest area (MWS) |  |  |
|  | Municipal point sources (MWS) | | [State Water Holding - Polish Waters: National Program for Urban Waste Water Treatment - https://www.wody.gov.pl/nasze-dzialania/krajowy-program-oczyszczania-sciekow-komunalnych   Statistics Poland: Local Data Bank. https://bdl.stat.gov.pl/BDL](https://www.wody.gov.pl/nasze-dzialania/krajowy-program-oczyszczania-sciekow-komunalnych) | National Program for Urban Waste Water Treatment - https://www.wody.gov.pl/nasze-dzialania/krajowy-program-oczyszczania-sciekow-komunalnych, Central Statistical Office - https://bdl.stat.gov.pl/BDL, State Water Holding-Polish Waters |
|  | Industrial point sources (INS) | | Statistics Poland: Local Data Bank. https://bdl.stat.gov.pl/BDL |  |
|  | Fertilization | | [State Water Holding - Polish Waters: "Identification of pressures in water regions and river basins districts. Part II: Development of database of anthropogenic pressures.  Statistics Poland: Local Data Bank. https://bdl.stat.gov.pl/BDL](https://bdl.stat.gov.pl/BDL) | Local Data Bank of the Statistics Poland (GUS) - https://bdl.stat.gov.pl/BDL/start with regard to the consumption of mineral fertilizers and livestock (for natural fertilizers) together with agrotechnical activities carried out in the catchment area. |
|  | Municipal diffuse sources (SCS) | | [Population: Statistics Poland - https://bdl.stat.gov.pl/BDL,  Unit load in untreated wastewater: State Water Holding - Polish Waters: National Program for Urban Waste Water Treatment - https://www.wody.gov.pl/nasze-dzialania/krajowy-program-oczyszczania-sciekow-komunalnych](https://www.wody.gov.pl/nasze-dzialania/krajowy-program-oczyszczania-sciekow-komunalnych) | National Program for Urban Waste Water Treatment - https://www.wody.gov.pl/nasze-dzialania/krajowy-program-oczyszczania-sciekow-komunalnych, Central Statistical Office - https://bdl.stat.gov.pl/BDL, State Water Holding-Polish Waters |
|  | Natural background (NBS) | | Calculated based on total outflow from catchment and concentrations of nutrients adopted from Polish PLC-7 report (SWH PW, 2020b). | [Chief Inspectorate for Environmental Protection in Poland. TN and TP loads from natural transformation and transport processes, based on land use. https://www.gios.gov.pl/en/](https://www.gios.gov.pl/en/) |
|  | Atmospheric deposition (ATS) | | Chief Inspectorate for Environmental Protection: Precipitation Chemistry (TN and TP). https://powietrze.gios.gov.pl | Chief Inspectorate for Environmental Protection: Precipitation Chemistry (TN) and dry deposition. https://powietrze.gios.gov.pl |

Table S2 The Wełna River model calibration, verification and validation results.

| **Calculation profile** | **Parameter** | **R^2^** | **PBIAS** | **KGE** |
| --- | --- | --- | --- | --- |
| **Calibration** | | | | |
| Flinta - Ryczywol | flow | 0.71 | -8 | 0.78 |
| Wełna - Prusce |  | 0.85 | -5 | 0.9 |
|  |  |  |  |  |
| Wełna - Oborniki | TN | 0.86 | -20 | 0.7 |
|  | TP | 0.60 | 30 | 0.53 |
| **Verification** | | | | |
| Flinta - Ryczywol | flow | 0.66 | 2 | 0.62 |
| Wełna - Prusce |  | 0.83 | 10 | 0.86 |
|  |  |  |  |  |
| Wełna - Oborniki | TN | 0.86 | 12.5 | 0.67 |
|  | TP | 0.38 | -25 | 0.21 |
| **Validation** | | | | |
| Wełna - Kowanowko | flow | 0.8 | 15 | 0.81 |
|  |  |  |  |  |
| Wełna - Rogoźno | TN | 0.9 | -14 | 0.51 |
|  | TP | 0.35 | 23 | 0.47 |

Table S3 Classification of value ranges for statistical measures used during calibration, verification, and validation^[3][4][5]^

| Performance Rating | **R^2^** | | | **PBIAS %** | | **KGE** |
| --- | --- | --- | --- | --- | --- | --- |
|  | flow | TN | TP | flow | TN/TP | flow/TN/TP |
| very good | > 0.85 | > 0.7 | > 0.8 | < 5 | < 15 | >0.75 |
| good | 0.75<R2<0.85 | 0.6<R2<0.7 | 0.65<R2<0.8 | 5<PBIAS<10 | 15<PBIAS<20 | 0.5 - 0.75 |
| satisfactory | 0.6<R2<0.75 | 0.3<R2<0.6 | 0.40<R2<0.65 | 10<PBIAS<15 | 20<PBIAS<30 | 0 - 0.5 |
| unsatisfactory | < 0.6 | < 0.3 | < 0.4 | > 15 | > 30 | <0 |

Table S4 R2 parameter values for additional water quality parameters - calibration period (2005-2009).

| Calculation profile | | Parameter |
| --- | --- | --- |
| Wełna - Oborniki | Mała Wełna - Rogoźno |  |
| R^2^ | |  |
| 0,55 | 0,04 | nitrite – N-NO_2_ |
| 0,83 | 0,88 | nitrate – N-NO_3_ |
| 0,27 | 0,55 | ammonium – N-NH_4_ |
| 0,13 | 0,10 | mineral phosphorus P-PO_4_ |

# References

1. Neitsch, S.L.; Arnold, J.G.; Kiniry, J.R.; Williams, J.R. 2011. Soil and Water Assessment Tool. Theoretical Documentation. Version 2009. *Texas Water Resources Institute. Technical Report No. 406. Texas A&M University System. College Station, Texas* 77843-2118
2. Betrie, G. D., Deng, B., Wang, J., Chang, M., & Al-Shamali, F. 2015. Integrated modeling of the Athabasca River Basin using SWAT. *Proceedings of Science and Technology Innovations, Faculty of Science and Technology, Athabasca University, Alberta, Canada,* 27-38.
3. Moriasi, D. N., Gitau, M. W., Pai, N., Daggupati, P. 2015. Hydrologic and water quality models: Performance measures and evaluation criteria. *Transactions of the ASABE*, **58(6**), 1763–1785. <http://dx.doi.org/10.13031/trans.58.10715>
4. Patil, S. D., Stieglitz, M. 2015. Comparing spatial and temporal transferability of hydrological model parameters. *Journal of Hydrology*: **525**, 409–417. <https://doi.org/10.1016/j.jhydrol.2015.04.003>
5. Libera, D. A., Sankarasubramanian, A. 2018. Multivariate bias corrections of mechanistic water quality model predictions. *Journal of Hydrology*: **564**, 529–541. <https://doi.org/10.1016/j.jhydrol.2018.07.043>
